# Impact of preoperative anemia on relapse and survival in breast cancer patients

**DOI:** 10.1186/1471-2407-14-844

**Published:** 2014-11-18

**Authors:** YingJun Zhang, YuYang Chen, DongTai Chen, Yu Jiang, Wan Huang, HanDong Ouyang, Wei Xing, MuSheng Zeng, XiaoMing Xie, Weian Zeng

**Affiliations:** Anesthesiology Department, State Key Laboratory in South China, Sun Yat-Sen University Cancer Center, 651 Dongfeng East Road, Guangzhou, PR China; Department of Cardiology, Sun Yat-sen Memorial Hospital of Sun Yat-Sen University, Guangzhou, PR China; State Key Laboratory in South China, Sun Yat-Sen University Cancer Center, Guangzhou, PR China; Department of Breast Cancer, Sun Yat-Sen University Cancer Center, Guangzhou, PR China

**Keywords:** Preoperative anemia, Breast cancer, Relapse, Survival, Hypoxia

## Abstract

**Background:**

Previous studies have shown that preoperative anemia is correlated with the prognoses of various solid tumors. This study was performed to determine the effect of preoperative anemia on relapse and survival in patients with breast cancer.

**Methods:**

A total of 2960 patients with breast cancer who underwent surgery between 2002 and 2008 at the Sun Yat-sen University Cancer Center (Guangzhou, PR China) were evaluated in a retrospective analysis. A total of 2123 qualified patients were divided into an anemic group [hemoglobin (Hb) < 12.0 g/dL, N = 535)] and a nonanemic group (Hb ≥ 12.0 g/dL, N = 1588). The effects of anemia on local relapse-free survival (LRFS), lymph node metastasis-free survival (LNMFS), distant metastasis-free survival (DMFS), relapse-free survival (RFS), and overall survival (OS) were assessed using Kaplan–Meier analysis. Independent prognostic factors were identified in the final multivariate Cox proportional hazards regression model.

**Results:**

Among the 2123 women who qualified for the analysis, 535 (25.2%) had a Hb level < 12.0 g/dL. The Kaplan–Meier curves showed that anemic patients had worse LRFS, LNMFS, DMFS, RFS, and OS than nonanemic patients, even in the same clinical stage of breast cancer. Cox proportional hazards regression model indicated that preoperative anemia was an independent prognostic factor of LRFS, LNMFS, DMFS, RFS, and OS for patients with breast cancer.

**Conclusions:**

Preoperative anemia was independently associated with poor prognosis of patients with breast cancer.

## Background

Anemia is a common complication in patients with cancer. It has been reported that between 30–90% of patients with cancer have anemia [1]. Most studies have found that pre-treatment anemia is associated with a worse prognosis in cancer patients [2–5]. In a meta-analysis, anemic patients with lung cancer, cervicouterine carcinoma, head and neck cancer, prostate cancer, lymphoma, and multiple myeloma had shorter survival times than those without anemia. The overall estimated increase in risk was 65% (54–77%) [6]. Preoperative anemia, even mild anemia, was independently associated with an increased risk of 30-day morbidity and mortality in patients undergoing major noncardiac surgery [7].

Breast cancer is one of the most common carcinomas worldwide among women. Tumor size, nodal status, histological grade, lymphovascular invasion (LVI), gene profile and Human Epidermal Growth Factor Receptor-2 (HER-2)-positivity are strong prognostic factors of breast cancer [8–10]. Although 41–82% of breast cancer patients develop anemia before surgery, [1] few studies have explored the effects of preoperative anemia on the prognosis of breast cancer. Whether preoperative anemia has a significant adverse impact on relapse or survival in breast cancer patients is still controversial [11, 12].

In this study, we aimed to determine the effects of preoperative anemia on relapse (local relapse, lymph node metastasis, distant metastasis, and overall relapse) and survival (local relapse-free survival, lymph node metastasis-free survival, distant metastasis-free survival, relapse-free survival, and overall survival) in patients undergoing breast cancer surgery.

## Methods

A total of 2960 patients with breast cancer who underwent surgery between 2002 and 2008 at the Sun Yat-sen University Cancer Center (Guangzhou, PR China) were evaluated in a retrospective analysis. This study was approved by the ethics committee of the Sun Yat-sen University Cancer Center. No consent from patients was needed.

**Figure 1:**
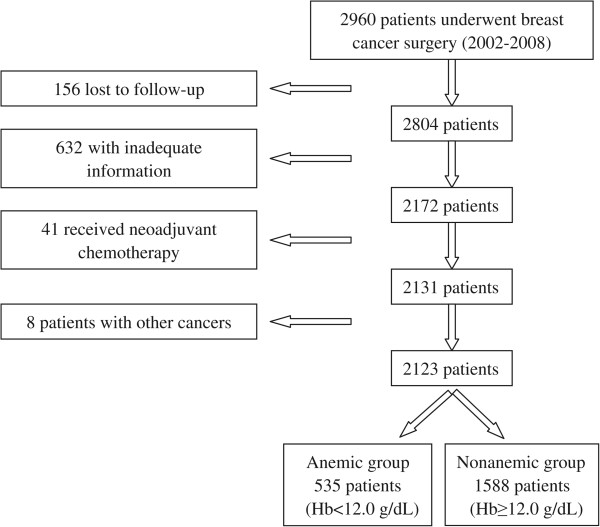
**Flow chart of the patient grouping.**

We defined the preoperative blood hemoglobin (Hb) concentration as the last Hb measurement before the index operation. We also collected other clinical data for subsequent analysis, including age, tumor type, tumor (T) and nodal (N) status, histological grade, estrogen receptor (ER) and progesterone receptor (PR) status, Human Epidermal Growth Factor Receptor-2 (Her-2) status, body mass index (BMI), menopausal status, type of surgery, and the use of chemotherapy, radiotherapy, endocrinotherapy, or targeted therapy. Patients with inadequate information, T_0_ stage cancer, metastases or inoperable tumors, as well as those treated with neoadjuvant chemotherapy or lost to follow-up were excluded from this analysis. Finally, 2123 patients were enrolled (Figure 1). We defined preoperative anemia as Hb < 12.0 g/dL and mild anemia as 9.0 ≤ Hb < 12.0 g/dL according to the World Health Organization (WHO) limits for Hb. The patients were divided into two groups based on this definition: the anemic patients group (Hb < 12.0 g/dL) and the nonanemic patient group (Hb ≥ 12.0 g/dL).

We defined local relapse-free survival (LRFS) as the duration from the surgery date to the date when local relapse was diagnosed. Lymph node metastasis-free survival (LNMFS) was defined as the duration from the surgery date to the date when lymph node metastasis was diagnosed. Distant metastasis-free survival (DMFS) was defined as the duration from the surgery date to the date when distant metastasis was diagnosed. Relapse-free survival (RFS) was defined as the duration from the surgery date to the date when any relapse was diagnosed and overall survival (OS) as the duration from the surgery date to the date of death or the last follow-up.

The clinical stages of breast cancer were performed according to the American Joint Committee on Cancer (AJCC) staging system [13]. Stage I included T_1_, N_0_, M_0_, stage II included IIA (T_0–1_, N_1_, M_0_ or T_2_, N_0_, M_0_) and IIB (T_2_, N_1_, M_0_ or T_3_, N_0_, M_0_) and stage III included IIIA (T_0–2_, N_2_, M_0_ or T_3_, N_1–2_, M_0_), IIIB (T_4_, N_0–2_, M_0_) and IIIC (any T, N_3_, M_0_). Stage IV was not considered because the patients with metastases were excluded.

### Statistical analysis

Patients’ characteristics (frequency distributions) were analyzed using the χ^2^ test (chi-squared test). Spearman rank correlation coefficients of risk factors for both anemia and nonanemia groups were determined. We also used the χ^2^ test to compare the local relapse, lymph node metastasis, distant metastasis, overall relapse, and mortality rates between the two groups. The comparison of LRFS, LNMFS, DMFS, RFS, and OS between anemic and nonanemic groups was performed using Kaplan–Meier analysis with the log-rank test. Multivariate Cox proportional hazards regression model with forward stepwise approach was constructed to identify independent prognostic factors. Age, tumor type, T-status, N-status, histologic grade, ER, PR, HER-2, BMI grade, menopause, type of surgery, anemia, sequential treatment after surgery (chemotherapy, radiotherapy, hormonal therapy, and targeted therapy) were predictive variables in the model. All statistical analyses were performed with SPSS (Statistical Package for the Social Sciences, IBM, NY, USA) version 16.0 software. A *P* value <0.05 was considered statistically significant.

## Results

Among a total of 2123 female patients qualified for the analysis, 535 (25.2%) had a Hb level < 12.0 g/dL. The median age of the patients was 47.0 (range, 22–91) years. There were 484 patients in stage I, 1198 in stage II, and 441 in stage III, and the corresponding number of anemic patients at each stage was 89 (18.4%), 283 (23.6%), and 163 (37.0%), respectively. Overall, 15.8% of the patients received locoregional radiotherapy, and 82.1% received adjuvant chemotherapy. Patient characteristics are shown in Table 1.

**Table 1:** **Clinical characteristics of patient by anemia status**

	***N*** = 2123 (%)	Hb < 12 g/dL	Hb ≥ 12 g/dL	***χ*** ^***2***^	***P***
		***n*** = 535 (25.2%)	***n*** = 1588 (74.8%)		
Age					
≤50	1384 (65.2)	359 (67.1)	1025 (64.5)	1.152	0.283
>50	739 (34.8)	176 (32.9)	563 (35.5)		
Tumor type					
Invasive ductal carcinoma	1944 (91.6)	503 (94.0)	1441 (90.7)	5.561	0.018
Other	179 (8.4)	32 (6.0)	147 (9.3)		
Tumor stage					
T1	703 (33.1)	146 (27.3)	557 (35.1)	32.458	<0.001
T2	1146 (54.0)	284 (53.1)	862 (54.3)		
T3 and T4	274 (12.9)	105 (19.6)	169 (10.6)		
N stage					
N0	1185 (55.8)	250 (46.7)	935 (58.8)	38.534	<0.001
N1	603 (28.4)	159 (29.7)	444 (28.0)		
N2	211 (9.9)	78 (14.6)	133 (8.4)		
N3	124 (5.8)	48 (9.0)	76 (4.8)		
Histologic grading					
G1G2 or Gx	1680 (79.1)	425 (79.4)	1255 (79.0)	0.041	0.840
G3	443 (20.9)	110 (20.6)	333 (21.0)		
ER					
Negative	846 (39.8)	226 (42.3)	620 (39.1)	6.385	0.041
Positive	683 (32.2)	182 (34.0)	501 (31.5)		
Strongly positive	594 (28.0)	127 (23.7)	467 (29.4)		
PR					
Negative	654 (30.8)	168 (31.4)	486 (30.6)	8.078	0.018
Positive	906 (42.7)	249 (46.5)	657 (41.4)		
Strongly positive	563 (26.5)	118 (22.1)	445 (28.0)		
HER-2					
Negative	1067 (50.3)	249 (46.5)	818 (51.5)	10.315	0.006
Positive	633 (29.8)	154 (28.8)	479 (30.2)		
Strongly positive	423 (19.9)	132 (24.7)	291 (18.3)		
BMI					
Low (<18.5)	151 (7.1)	47 (8.8)	104 (6.6)	25.980	<0.001
Normal (18.5–22.9)	929 (43.8)	276 (51.6)	653 (41.1)		
High (>22.9)	1043 (49.1)	212 (39.6)	831 (52.3)		
Menopause					
No	1318 (62.1)	352 (65.8)	966 (60.8)	4.188	0.041
Yes	805 (37.9)	183 (34.2)	622 (39.2)		
Type of surgery					
Modified radical mastectomy	2092 (98.5)	531 (99.3)	1561 (98.3)	2.524	0.112
Breast-conserving surgery	31 (1.5)	4 (0.7)	27 (1.7)		
Chemotherapy					
No	381 (17.9)	89 (16.6)	292 (18.4)	0.835	0.361
Yes	1742 (82.1)	446 (83.4)	1296 (81.6)		
Radiotherapy					
No	1842 (86.8)	452 (84.5)	1390 (87.5)	3.232	0.072
Yes	281 (13.2)	83 (15.5)	198 (12.5)		
Hormonal therapy					
No	1366 (64.3)	347 (64.9)	1019 (64.2)	0.083	0.773
Yes	757 (35.7)	188 (35.1)	569 (35.8)		
Targeted therapy					
No	2109 (99.3)	530 (99.1)	1579 (99.4)	-	0.361^a^
Yes	14 (0.7)	5 (0.9)	9 (0.6)		

^a^Fisher's exact test.

*Abbreviations: Hb* hemoglobin, *PR* partial response, *BMI* body mass index.

The relation between Hb levels and various risk factors was examined by Spearman rank correlation coefficients. As shown in Table 2, we found that there was a significant positive correlation between Hb levels and BMI, and a negative correlation with T- and N-status and clinical stages.

**Table 2:** **Spearman’s rank correlation of the hemoglobin levels and various clinical characteristics**

	Hb	***P***
Age	0.035	0.101
Tumor type	0.014	0.509
T stage	−0.078	<0.001
N stage	−0.051	0.019
Clinical stage	−0.085	<0.001
Histologic grading	0.010	0.653
ER	0.029	0.181
PR	0.016	0.460
HER-2	−0.035	0.103
BMI	0.134	<0.001
Chemotherapy	−0.025	0.242
Radiotherapy	−0.014	0.521
Hormonal therapy	0.002	0.912
Targeted therapy	−0.034	0.115

*Abbreviations: Hb* hemoglobin, *ER* estrogen receptor, *PR* progesterone receptor, *HER-2* Human Epidermal Growth Factor Receptor-2, *BMI* body mass index.

After a median follow-up time of 67 months, 61 patients (2.9%) underwent local relapse, 105 (4.9%) had lymph node metastases, and 269 (12.7%) had distant metastases among 2123 breast cancer patients. Local relapse was diagnosed in 7.3% of anemic patients versus 1.4% of nonanemic patients (*P* < 0.001). For lymph node metastasis, distant metastasis, and any relapse, the percentages were 12.1% versus 2.5% (*P* < 0.001), 26.7% versus 7.9% (*P* < 0.001) and 38.7% versus 9.9% (*P* < 0.001), respectively. Mortality was 24.5% in anemic group versus 7.7% in nonanemic group (*P* < 0.001) (Table 3). The relapse rate and mortality were significantly different between the anemic and nonanemic groups.

**Table 3:** **Prevalence of relapses and deaths in patients with and without anemia**

	***N*** = 2123	Hb < 12 g/dL	Hb ≥ 12 g/dL	***χ*** ^2^	***P***
		***n*** = 535 (%)	***n*** = 1588 (%)		
Local relapse					
No	2062	496 (92.7)	1566 (98.6)	49.989	<0.001
Yes	61	39 (7.3)	22 (1.4)		
Lymph node metastasis					
No	2018	470 (87.9)	1548 (97.5)	78.950	<0.001
Yes	105	65 (12.1)	40 (2.5)		
Distant metastasis					
No	1854	392 (73.3)	1462 (92.1)	127.7	<0.001
Yes	269	143 (26.7)	126 (7.9)		
Any relapse					
No	1758	328 (61.3)	1430 (90.1)	232.2	<0.001
Yes	365	207 (38.7)	158 (9.9)		
Death					
No	1869	404 (75.5)	1465 (92.3)	106.5	<0.001
Yes	254	131 (24.5)	123 (7.7)		

*Abbreviation:* *Hb* hemoglobin.

**Figure 2:**
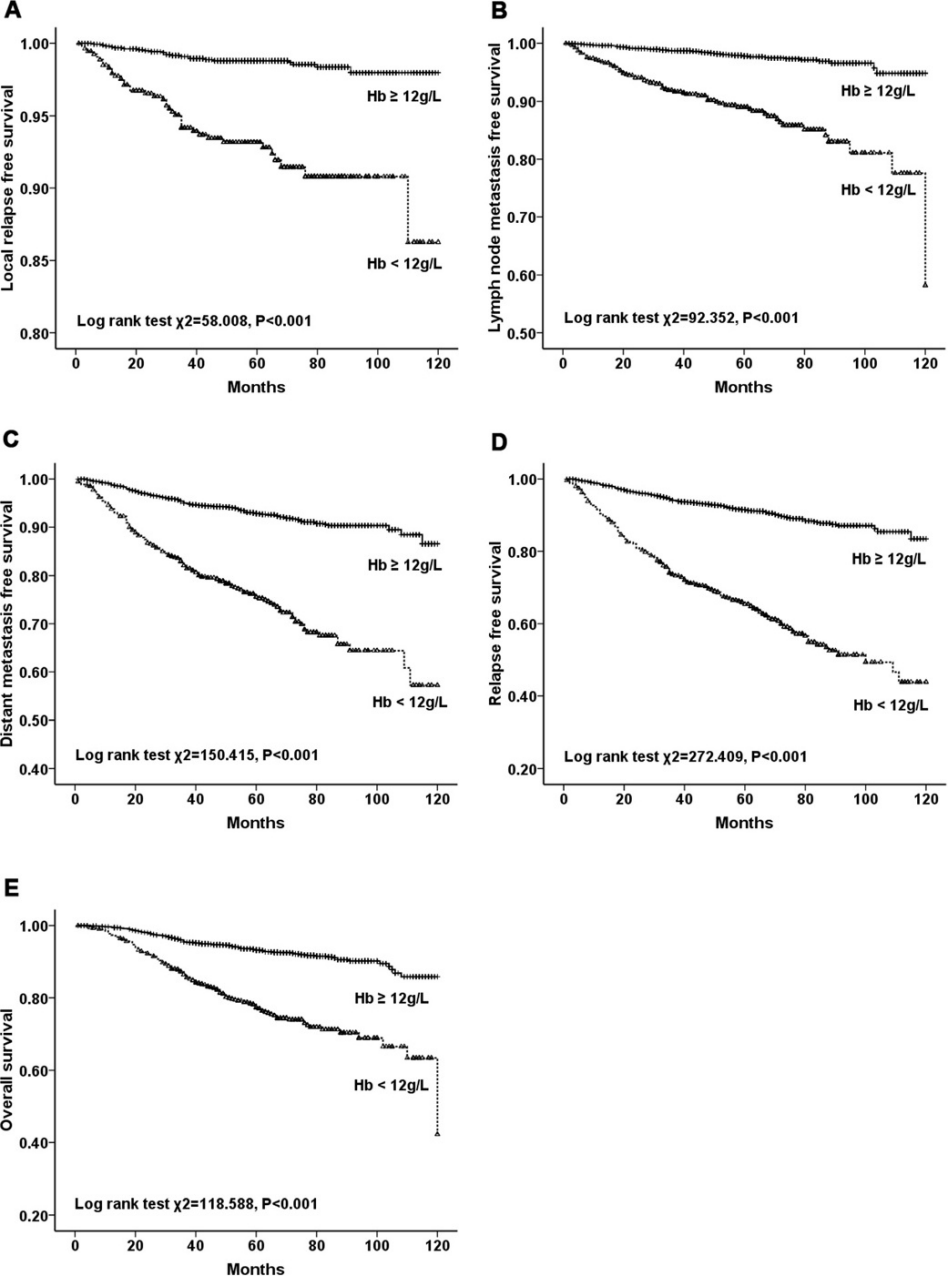
**LRFS, LNMFS, DMFS, RFS, and OS of patients with and without anemia. A**. LRFS for patients with Hb ≥ 12 g/dL versus Hb < 12 g/dL. **B**. LNMFS for patients with Hb ≥12 g/dL versus Hb <12 g/dL. **C**. DMFS for patients with Hb ≥12 g/dL versus Hb <12 g/dL. **D**. RFS for patients with Hb ≥12 g/dL versus Hb <12 g/dL. **E**. OS for patients with Hb ≥12 g/dL versus Hb <12 g/dL.

**Figure 3:**
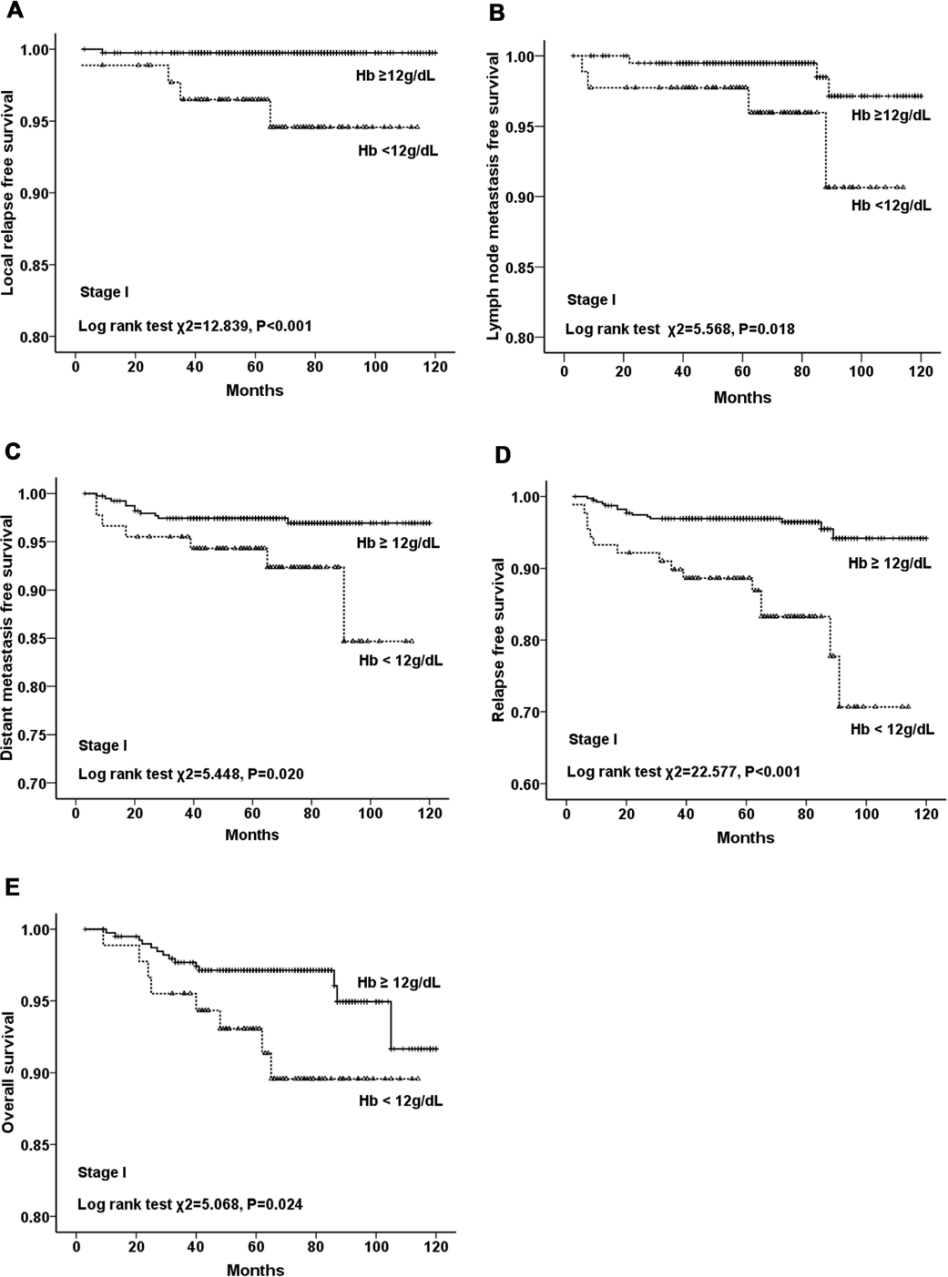
**LRFS, LNMFS, DMFS, RFS, and OS of patients in stage I with and without anemia. A**. LRFS for patients with Hb ≥12 g/dL versus Hb <12 g/dL in stage I. **B**. LNMFS for patients with Hb ≥12 g/dL versus Hb <12 g/dL in stage I. **C**. DMFS for patients with Hb ≥12 g/dL versus Hb <12 g/dL in stage I. **D**. RFS for patients with Hb ≥12 g/dL versus Hb <12 g/dL in stage I. **E**. OS for patients with Hb ≥12 g/dL versus Hb <12 g/dL in stage I.

**Figure 4:**
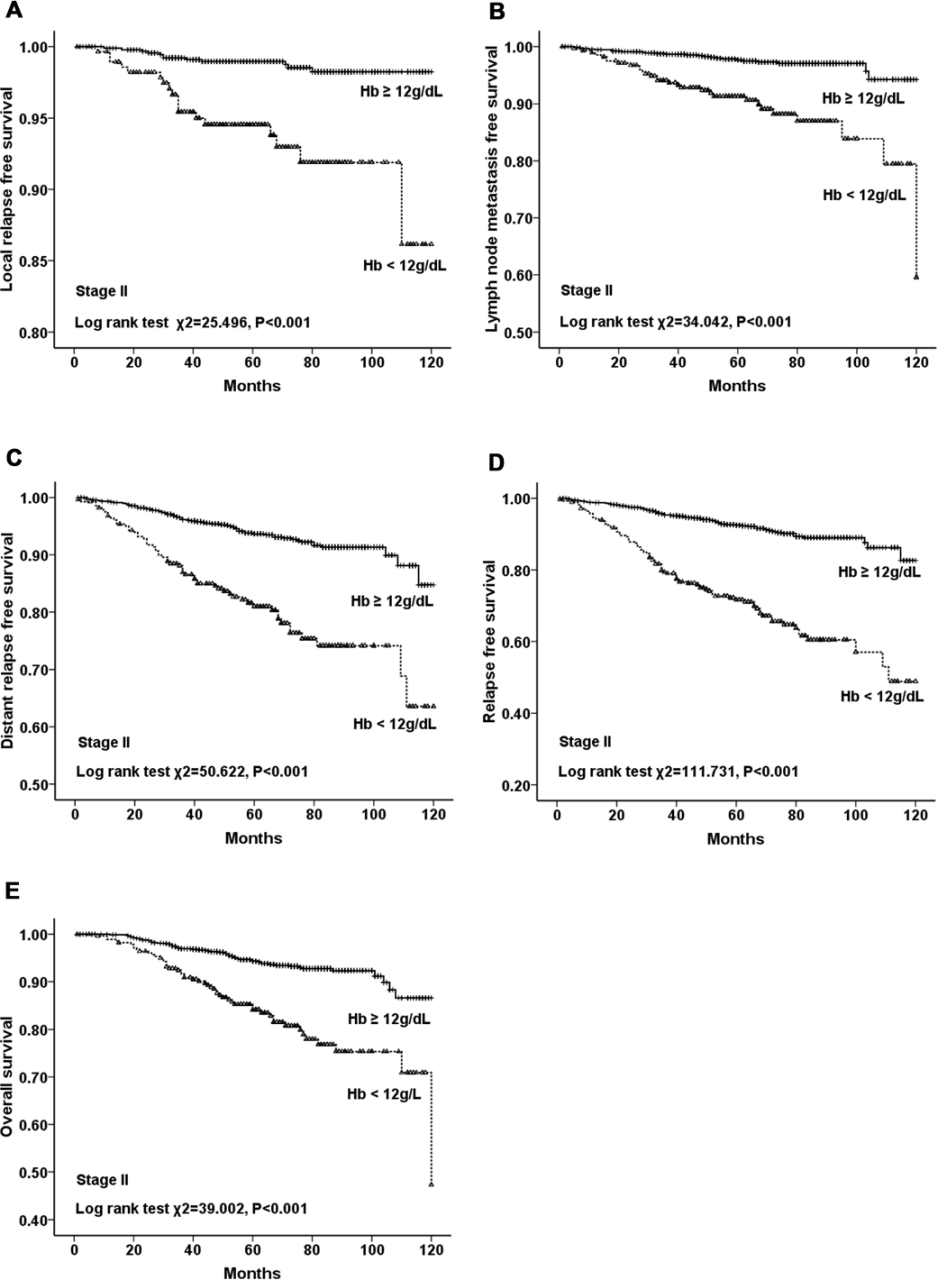
**LRFS, LNMFS, DMFS, RFS, and OS for patients in stage II with and without anemia. A**. LRFS for patients with Hb ≥12 g/dL versus Hb <12 g/dL in stage II. **B**. LNMFS for patients with Hb ≥12 g/dL versus Hb <12 g/dL in stage II. **C**. DMFS for patients with Hb ≥12 g/dL versus Hb <12 g/dL in stage II. **D**. RFS for patients with Hb ≥12 g/dL versus Hb <12 g/dL in stage II. **E**. OS for patients with Hb ≥12 g/dL versus Hb <12 g/dL in stage II.

**Figure 5:**
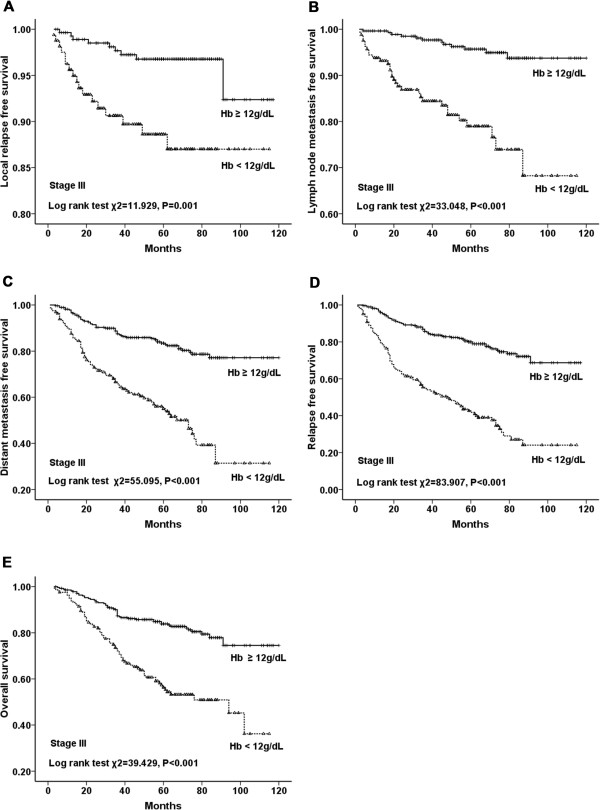
**LRFS, LNMFS, DMFS, RFS, and OS for patients in stage III with and without anemia. A**. LRFS for patients with Hb ≥12 g/dL versus Hb <12 g/dL in stage III. **B**. LNMFS for patients with Hb ≥12 g/dL versus Hb <12 g/dL in stage III. **C**. DMFS for patients with Hb ≥12 g/dL versus Hb <12 g/dL in stage III. **D**. RFS for patients with Hb ≥12 g/dL versus Hb <12 g/dL in stage III. **E**. OS for patients with Hb ≥12 g/dL versus Hb <12 g/dL in stage III.

**Figure 6:**
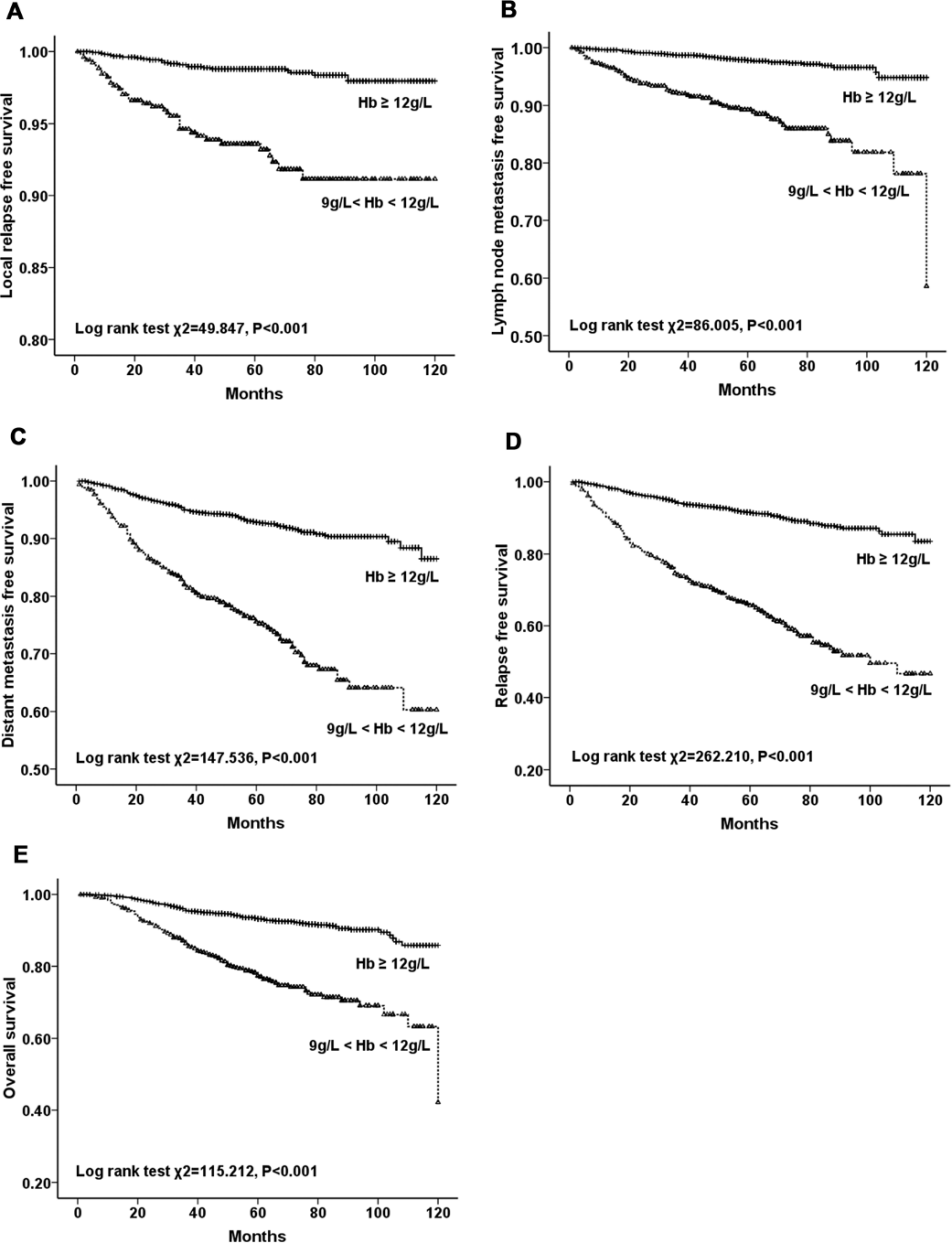
**LRFS, LNMFS, DMFS, RFS, and OS for patients without anemia versus mild anemia. A**. LRFS for patients with Hb ≥12 g/dL versus 9 < Hb <12 g/dL. **B**. LNMFS for patients with Hb ≥12 g/dL versus 9 < Hb <12 g/dL. **C**. DMFS for patients with Hb ≥12 g/dL versus 9 < Hb <12 g/dL. **D**. RFS for patients with Hb ≥12 g/dL versus 9 < Hb <12 g/dL. **E**. OS for patients with Hb ≥12 g/dL versus 9 < Hb <12 g/dL.

In the univariate analysis, LRFS, LNMFS, DMFS, RFS, and OS were significantly shorter in anemic patients than those in nonanemic patients (*P* < 0.001 for all) (Figure 2). Additionally, stratified analysis by different clinical stages (stages I to III) of breast cancer showed that LRFS, LNMFS, DMFS, RFS and OS were all significantly shorter in anemic patients (Figures 3, 4 and 5). Among the 2123 anemic patients, 2104 had mild anemia (9.0 ≤ Hb < 12.0 g/dL). Survivals were also significantly shorter even in patients with mild anemia (Figure 6).

Multivariate analysis with all relevant prognostic factors in a Cox proportional hazards regression model showed that preoperative anemia was a significant prognostic factor in breast cancer patients (Table 4). T-status (≥T_3_), N-status (N_1_, N_2_), strongly positive PR status and HER-2 positivity were significantly associated with LRFS, and anemic patients had a 4.939-fold increased relative risk of developing local relapse compared with nonanemic patients. Only the N-status (N_1_, N_2_) was significantly associated with LNMFS, with a 5.160-fold increased relative risk of developing lymph node metastasis for anemic patients compared with nonanemic patients. With respect to DMFS and OS, T-status (≥T_3_) and N-status (N_1_-N_3_) still had significant associations, and the relative risks of developing distant metastasis and death in the anemic group were 3.192-fold and 2.849-fold higher than those in the nonanemic group, respectively. For RFS, T-status (≥T_3_), N-status (N_1_–N_3_), and strongly positive PR status were shown to be significant prognostic factors. Anemic patients had a 4.104-fold increased relative risk of developing any relapse compared with nonanemic patients.

**Table 4:** **Multivariate analysis of prognostic factors for LRFS, LNMFS, DMFS, RFS, and OS**

	LRFS	LNMFS	DMFS	RFS	OS					
	HR (95% CI)	***P***	HR (95% CI)	***P***	95% CI	***P***	95% CI	***P***	95% CI	***P***
T stage										
T_1_	*Ref*		NS	NS	*Ref*		*Ref*		*Ref*	
T_2_	1.045 (0.532–2.050)	0.899	NS	NS	1.333 (0.962–1.847)	0.084	1.287 (0.976–1.697)	0.074	1.291 (0.925–1.803)	0.134
≥T_3_	2.676 (1.267–5.653)	0.010	NS	NS	1.983 (1.347–2.920)	0.001	2.021 (1.455–2.807)	<0.001	2.020 (1.371–2.975)	<0.001
N stage										
N_0_	*Ref*		*Ref*		*Ref*		*Ref*		*Ref*	
N_1_	2.601 (1.366–4.963)	0.004	2.235 (1.366–3.657)	0.001	2.040 (1.493–2.788)	<0.001	2.009 (1.544–2.615)	<0.001	1.942 (1.404–2.687)	<0.001
N_2_	2.708 (1.122–6.534)	0.027	3.742 (2.058–6.805)	<0.001	3.484 (2.358–5.147)	<0.001	3.016 (2.152–4.225)	<0.001	4.200 (2.854–6.181)	<0.001
N_3_	2.450 (0.859–6.989)	0.094	2.045 (0.912–4.487)	0.083	4.822 (3.175–7.323)	<0.001	3.856 (2.672–5.565)	<0.001	5.083 (3.307–7.812)	<0.001
ER										
Negative	*Ref*		NS	NS	*Ref*		*Ref*		*Ref*	
Positive	0.525 (0.261–1.057)	0.071	NS	NS	0.670 (0.479–0.937)	0.019	0.726 (0.547–0.965)	0.027	0.845 (0.598–1.194)	0.340
Strongly positive	0.340 (0.144–0.803)	0.014	NS	NS	0.804 (0.537–1.206)	0.292	0.757 (0.534–1.074)	0.119	0.566 (0.360–0.890)	0.014
PR										
Negative	*Ref*		NS	NS	NS	NS	*Ref*	NS	NS	NS
Positive	1.709 (0.826–3.535)	0.149	NS	NS	NS	NS	1.409 (1.066–1.861)	0.016	NS	NS
Strongly positive	2.989 (1.236–7.228)	0.015	NS	NS	NS	NS	0.899 (0.611–1.322)	0.588	NS	NS
HER-2										
Negative	*Ref*		NS	NS	NS	NS	NS	NS	NS	NS
Positive	2.179 (1.232–3.855)	0.007	NS	NS	NS	NS	NS	NS	NS	NS
Strongly positive	0.651 (0.292–1.451)	0.294	NS	NS	NS	NS	NS	NS	NS	NS
Hormonal therapy	NS	NS	0.537 (0.335–0.859)	0.009	NS	NS	0.733 (0.575–0.933)	0.012	0.682 (0.503–0.926)	0.014
Anemia	4.939 (2.875–8.484)	<0.001	5.160 (3.428–7.767)	<0.001	3.192 (2.489–4.094)	<0.001	4.104 (3.310–5.089)	<0.001	2.849 (2.205–3.680)	<0.001

*Abbreviations:* *LRFS* local relapse-free survival, *LNMFS* lymph node metastasis-free survival, *DMFS* distant metastasis-free survival, *RFS* relapse-free survival, *OS* overall survival, *ER* estrogen receptor, *PR* progesterone receptor, *HER-2* Human Epidermal Growth Factor Receptor-2, *HR* hazard ration, *CI* confidence interval, *Ref*: Reference group; NS: No significance.

## Discussion

Preoperative anemia has been reported to be associated with poor prognosis in many types of tumors [6, 14]. In our present study, a low preoperative Hb level was shown to be associated with local and distant relapses in breast cancer patients. Shorter survival was also observed in anemic patients. To the best of our knowledge, our study was the first to discover that preoperative Hb levels were associated with tumor (T) and nodal (N) status of breast cancer and BMI. Further, the most important study finding was that preoperative anemia was shown to be an independently prognostic factor for LRFS, LNMFS, DMFS, RFS, and OS in breast cancer patients, even in the same clinical stage or at lower stages.

Causes of anemia in cancer patients are multifactorial and can be considered as results of cancer invasion, induced by treatment (after radiotherapy or chemotherapy), or chronic kidney disease [15]. Among the three factors mentioned above, the first one is the largest contributor. Cancer itself can cause or exacerbate anemia in several ways [16]. Cancer cells may suppress hematopoiesis via bone marrow infiltration directly. They also generate cytokines that lead to functional iron deficiency, which decreases the production and shorten the survival of red blood cells [17]. Also, chronic blood loss at tumor sites through cancer cells infiltration can exacerbate anemia. Other indirect effects include nutritional deficiencies of iron, folate, and vitamin B12 secondary to anorexia or hemolysis by immune-mediated antibodies. For the factors mentioned above, it is plausible that preoperative anemia is more frequent in higher clinical stages and low BMI in association with malnutrition.

Many studies supported that pre-treatment Hb levels during adjuvant or neoadjuvant chemotherapy were related to the prognosis of breast cancer. However, few studies focused on the preoperative Hb levels [12, 18, 19]. Kandemir et al. reported that preoperative anemia was an independent risk factor of disease-free survival and overall survival in 336 early-stage breast cancer patients [11]. Our results not only supported their conclusion but also showed that preoperative anemia was associated with local relapse-free survival, lymph node metastasis-free survival, and distant metastasis-free survival in a larger cohort.

There are several possible mechanisms by which anemia may reduce survival, and hypoxia is the most important one. Anemia can reduce the capacity of the blood to transport oxygen (O_2_), further contributing to the development of hypoxia. Hypoxia is a common characteristic of locally advanced solid tumors that has been associated with greater recurrence, less locoregional control, diminished therapeutic responses, and lower overall and disease-free survival [20, 21]. The association between the blood Hb concentration (cHb) and the tumor oxygenation status has been examined [22–27]. The median pO_2_ values in breast cancer tumors are lower than those in the normal breast, which exponentially increase with increasing cHb values [28]. In normal breast tissue, the O_2_ tensions are approximately at a mean pO_2_ of 65 mmHg. However, in breast cancer tissue, the median pO_2_ is 28 mmHg. Further, nearly 60% of breast cancers contain hypoxic tissue areas with pO_2_ values <2.5 mmHg [29].

Hypoxia can lead to structural and functional abnormalities in the tumor microvasculature, an adverse diffusion geometry and tumor-related anemia result in a reduced O_2_ transport capacity of the blood [30]. A key regulator of this process is hypoxia-inducible factor-1 (HIF-1). HIF-1 is a molecular determinant that responds to hypoxia. Its expression increases as the pathologic stages progress, and it is higher in poorly differentiated lesions than in well-differentiated lesions [31]. HIF-1 activity mediates angiogenesis [32–34], epithelial-mesenchymal transition [25], genetic mutations, resistance to apoptosis, and resistance to radiotherapy and chemotherapy [34] in regions of intratumoral hypoxia. More recent studies have suggested that HIF-1α is a significant positive regulator of tumor progression, metastasis, and poor patient prognosis [26, 32, 33], and higher expression of HIF-1α has been shown to correlate with poorer survival in breast cancer patients [35, 36]. This effect was independent of standard prognostic factors, such as tumor stage and nodal status [37]. Some results of our study may be attributed to hypoxia and HIF-1α activity. It was interesting that preoperative Hb levels were negatively related to tumor (T) and nodal (N) status of breast cancer, which were both traditional prognostic factors of breast cancer. However, anemia also impaired various survival outcomes independently even in the same clinical stage.

Although preoperative anemia was not related to the sequential postoperative treatment in our study, most of the data supported the notion that pretreatment anemia may influence the effects of sequential postoperative treatment. The reason may be that preoperative anemia contributes to hypoxia in cancer cells. There is increasing evidence that hypoxic cancer cells are likely to be resistant to radiotherapy, chemotherapy, and targeted therapy. Thus, the potential for invasion, metastasis and patient mortality is increased further [25–27, 30]. Hypoxia leads to therapeutic resistance directly through a lack of O_2_, which radiation and some chemotherapeutic drugs require to exert their cytotoxicity. Hypoxia also leads to resistance indirectly through changes in cellular metabolism, proliferation kinetics, the cell-cycle position, the hypoxia-driven proteome, and genome and clonal selection [21, 27].

Although hypoxia may be a reasonable explanation for the association between anemia and survival of breast cancer, there was no direct evidence of hypoxia in cancer cells in our large population study. Emerging new tools that can measure the local Hb level and O_2_ tension directly in tumor tissues may solve this problem in the future. Our study provided a clue for further investigations to clarify the complex mechanisms of hypoxia in breast cancer.

Since preoperative anemia was associated with poor prognosis in breast cancer patients in our study, would patients benefit from anemia treatment preoperatively? Or could we improve the prognosis after administering treatment for anemia? The answer to this question is somewhat ambiguous because of the complexity of anemia. For most of patients with breast cancer without chemotherapy, preoperative anemia was caused by multiple etiologies, including blood loss, functional iron deficiency, erythropoietin deficiency secondary to renal disease, tumoral marrow involvement, well as other factors. Evaluation of anemia should be performed carefully before treatment because an unsuitable treatment might lead to adverse effects. The most common treatment options for anemic patients include iron therapy, red cell transfusion, and erythropoietic-stimulating agents. For iron therapy, nutritional status (iron, total iron binding capacity, ferritin, transferrin saturation, folate, and vitamin B_12_) and renal function should be evaluated. Only absolute iron deficiency will benefit from intravenous or oral iron monotherapy [38, 39]. Unfortunately the absence of data regarding the nutritional status and renal function of our patients impeded further analysis.

Red cell transfusion is an acceptable treatment option for anemic breast cancer patients, especially for those requiring rapid improvement of Hb levels. However, large-scale studies involving cancer patients found that red cell transfusion was associated with increased thrombosis risk as well as increased mortality risk [40]. Additionally, mild anemia accounted for 99% anemic patients in this study; thus, transfusions might not be necessary. As for erythropoietic-stimulating agent therapy, it was suitable only for patients receiving palliative, myelosuppressive chemotherapy with a Hb <10 g/dL and without absolute iron deficiency [39]. Notably, there were few reports focusing on the relationship between preoperative Hb and prognosis. However, most treatments for anemia were derived from the prognostic outcomes of patients with chemotherapy-induced anemia. Thus, whether preoperative anemia and chemotherapy-induced anemia are both associated with poor prognosis of patients with breast cancer remains to be clarified. The question of what is the best approach for patients with preoperative anemia remains unanswered. Therefore, further studies will be needed to answer these questions.

## Conclusions

Preoperative anemia is a negative prognostic factor for survival of patients with breast cancer. However, it still merits further experimental and clinical investigations.

## Abbreviations

LRFS: Local relapse free survival
LNMFS: Lymph node metastasis free survival
DMFS: Distance metastasis free survival
RFS: Relapse free survival
OS: Overall survival
LVI: Lymphovascular invasion
HER-2: Human epidermal growth factor receptor-2
Hb: Hemoglobin
TNM: Tumor-Node-Metastasis
ER: Estrogen receptor
PR: Progesterone receptor
BMI: Body mass index
WHO: World Health Organization
cHb: Blood hemoglobin concentration
HIF-1: Hypoxia-inducible factor-1.
